# An assessment of the impact of host polymorphisms on *Plasmodium falciparum var*gene expression patterns among Kenyan children

**DOI:** 10.1186/1471-2334-14-524

**Published:** 2014-09-29

**Authors:** George M Warimwe, Gregory Fegan, Esther W Kiragu, Jennifer N Musyoki, Alexander W Macharia, Kevin Marsh, Thomas N Williams, Peter C Bull

**Affiliations:** Kenya Medical Research Institute-Wellcome Trust Research Programme, Kilifi, Kenya; The Jenner Institute, University of Oxford, Oxford, UK; Nuffield Department of Clinical Medicine, University of Oxford, Oxford, UK; Department of Medicine, Imperial College, London, UK

**Keywords:** Malaria, Host polymorphisn, Parasite *var* gene

## Abstract

**Background:**

Host genotype accounts for a component of the variability in susceptibility to childhood *Plasmodium falciparum* malaria. However, despite numerous examples of host polymorphisms associated with tolerance or resistance to infection, direct evidence for an impact of host genetic polymorphisms on the *in vivo* parasite population is difficult to obtain. Parasite molecules whose expression is most likely to be associated with such adaptation are those that are directly involved in the host-parasite interaction. A prime candidate is the family of parasite *var* gene-encoded molecules on *P. falciparum*-infected erythrocytes, PfEMP1, which binds various host molecules and facilitates parasite sequestration in host tissues to avoid clearance by the spleen.

**Methods:**

To assess the impact of host genotype on the infecting parasite population we used a published parasite *var* gene sequence dataset to compare *var* gene expression patterns between parasites from children with polymorphisms in molecules thought to interact with or modulate display of PfEMP1 on the infected erythrocyte surface: ABO blood group, haemoglobin S, alpha-thalassaemia, the T188G polymorphism of CD36 and the K29M polymorphism of ICAM1.

**Results:**

Expression levels of ‘group A-like’ *var* genes, which encode a specific group of PfEMP1 variants previously associated with low host immunity and severe malaria, showed signs of elevation among children of blood group AB. No other host factor tested showed evidence for an association with *var* expression.

**Conclusions:**

Our preliminary findings suggest that host ABO blood group may have a measurable impact on the infecting parasite population. This needs to be verified in larger studies.

**Electronic supplementary material:**

The online version of this article (doi:10.1186/1471-2334-14-524) contains supplementary material, which is available to authorized users.

## Background

Host genetic factors have been estimated to account for up to 30% of the variability in the risk of developing malaria [1]. Whereas numerous studies have examined the effect of specific genetic polymorphisms on disease susceptibility and severity, relatively little attention has been paid to their direct impact on parasite gene expression patterns within the host. Such an effect may be most pronounced on parasite molecules that play a direct role in the host-parasite interaction. The large family of cytoadhesive parasite molecules called PfEMP1 (*P. falciparum* erythrocyte membrane protein 1) fit this profile. These multi-domain proteins are encoded by ~60 clonally variant *var* genes that are present within the subtelomeric regions of most chromosomes and within some internal clusters. PfEMP1 are inserted into the infected erythrocyte (IE) surface where they bind to various host ligands resulting in IE sequestration in different body tissues [2]. Sequestration of IE in tissues is thought to prevent their passage through the spleen, where they may be effectively removed from circulation. During the blood stage of infection by *P. falciparum*, individual parasites switch between different *var* genes through an epigenetic mechanism that operates at least in part at the transcriptional level. Through this process they can alter both the cytoadhesive and antigenic properties of infected erythrocytes and thus prolong infection [3, 4]. We would predict that expression of PfEMP1 by the infecting parasite population is very sensitive to both alterations in the composition of host receptors and in the composition of the erythrocyte surface itself. We and others have previously shown that host immunity imposes a selection pressure on the infecting parasite population such that a specific group of PfEMP1 variants are preferentially expressed in young children and those with a poorly developed repertoire of IE surface antibodies [5–7]. However, whether host genotype influences PfEMP1 expression patterns in an infecting parasite population remains unknown. This could occur, first, through host polymorphisms that alter the concentration of specific PfEMP1 ligands in the body. Within this scenario, polymorphisms in one host molecule may select against expression of PfEMP1 variants requiring that molecule for cytoadhesion. Parasites expressing such variants would therefore be left vulnerable to clearance by the spleen while those expressing PfEMP1 variants that can utilize other host molecules for cytoadhesion will evade splenic clearance and dominate the infection. Second, modification of PfEMP1 expression could occur if genetic variants accelerate the rate at which antibodies to the surface of IEs are acquired (this has been described for genetic variants of haemoglobin; [8–10]). Given that host IE surface antibodies are associated with parasite *var* gene expression patterns *in vivo*[7], differences in the host-PfEMP1 interaction with respect to such genotypes may be expected.

Here, to investigate the influence of host genotype on the infecting parasite population we compared *var* gene expression patterns among clinical isolates from Kenyan children in relation to: 1) three host polymorphisms in molecules to which PfEMP1 binds (namely, the T188G polymorphism of CD36 [11], the K29M polymorphism of ICAM1 [12] and ABO blood group [13]) and, 2) two host genetic variants of haemoglobin that accelerate the rate at which antibodies to the surface of IEs are acquired, alpha-thalassaemia and HbS [8–10].

## Methods

### Study site and participants

We used a published dataset of children presenting to hospital with clinical malaria (N = 217) and asymptomatic infections (N = 33) for all the analysis presented here [7, 14]. This dataset was specifically designed to evaluate the relationship between parasite *var* gene expression patterns and immunity to clinical malaria as described [7, 14]. However various host genotype data were also available for most children thus allowing an exploratory analysis on the effect of host genotype on parasite *var* gene expression patterns. The study was carried out at Kilifi District Hospital, situated at the coast of Kenya, following ethical approval by the Kenya Medical Research Institute Ethical Review Committee. Informed consent was obtained from parents/guardians of all study participants.

Clinical malaria was defined as fever plus ≥ 1 or more trophozoites per 100 uninfected erythrocytes [7, 14]. Severe malaria was defined as a hospital admission with any of the three major clinical syndromes that tend to be observed in African children [15, 16] namely: severe malarial anemia (haemoglobin concentration of <5 g/dL), impaired consciousness (Blantyre coma score <4 in patients under 8 months old or <5 in patients aged ≥ 8 months) and respiratory distress (deep ‘Kussmaul’ breathing pattern). The clinical malaria patients were sampled between August 2003 and September 2007 while children with asymptomatic infection of any parasite density were sampled in May 2007 during a population based cross-sectional survey.

### Host genotyping

Published methods were used for host genotyping of the T188G polymorphism of CD36 and K29M polymorphism of ICAM1 [17], alpha-thalassaemia [18] and HbAS [19]. ABO blood group for each patient was determined using standard slide haemagglutination.

### Parasite sampling and *var*sequence classification

Expressed *var* sequence tags were generated from cDNA obtained from ring-stage parasite isolates obtained from all 250 children before culture using published methods [6]. PCR amplified *var* tags were ligated into plasmids and sub-cloned into bacteria. For each parasite isolate, up to 96 individual bacterial colonies were picked, and plasmid DNA prepared for capillary sequencing. Sequence assembly, classification and counting were then done using two published analysis pipelines [7, 20]. For each parasite isolate the number of bacterial colonies carrying cys2 *var* sequences and each of three previously described cys2 sequence subgroups i.e. MFK + REY-, MFK-REY + and MFK-REY- [6] was expressed as a percentage of the total number of colonies sequenced and this proportion was used for the analysis. Using another published classification approach we also classified cys2 sequences as “group A-like” if they contained any of a set of 573 polymorphic sequence blocks [7, 21]. For each parasite isolate the number of colonies carrying group A-like sequences was also expressed as a percentage of the number of colonies sequenced.

### Statistical analysis

Analysis was performed using Stata™ statistical software version 11. Before use in linear regression models the percentage sequence expression levels were arcsine-transformed as described previously [7, 14]. Linear regression analysis was performed with binary variables for each blood group phenotype, that is, blood groups A, B, AB and O, with the latter used as the reference for comparison. For all other host molecules, the respective allelic states (i.e. heterozygous and homozygous) were used simultaneously as explanatory variables so that the results are relative to the wild type genotype (Table 1). We tested for an association between each polymorphism and expression levels of five markers of the parasite *var* gene expression profile that tend to be differentially associated with host immunity: i) the overall proportion of expressed *var* gene sequences that are classified as cys2 and, ii) the proportion of cys2 *var* genes that fall in the MFK + REY-, iii) MFK-REY+, iv) MFK-REY- or v) group A-like subgroup. Our aim was to determine whether host polymorphism have an independent effect on *var* gene expression and hence all regression models were adjusted for covariates previously found to be associated with cys2 *var* gene expression levels in the primary publications on this dataset namely: host age, IE surface antibody levels (measured as the median IgG response to the IE surface of 8 clinical isolates by flow cytometry) and infection type at sampling (coded as binary variables for either mild malaria, malaria with impaired consciousness, malaria with respiratory distress, severe malarial anemia or asymptomatic infection) [7, 14]. The validity of the observed relationships was confirmed by checking the normality of the residuals from the regression models. The interquartile ranges of the residuals had no severe outliers thus providing no evidence to reject normality of the residuals at a 5% alpha level.

**Table 1:** **Relationship between host genotype and parasite** ***var*** **gene expression**

		Cys2 ***var***group	Group A-like subgroup	MFK + REY- subgroup	MFK-REY + subgroup	MFK-REY- subgroup
Host polymorphism	Type, no. in dataset	Beta (95% CI), ***p***value	Beta (95% CI), ***p***value	Beta (95% CI), ***p***value	Beta (95% CI), ***p***value	Beta (95% CI), ***p***value
ABO blood group	Blood group O, 108	0	0	0	0	0
	Blood group A, 76	0.004 (-0.10, 0.11), *p* = 0.9	-0.004 (-0.10, 0.09), *p* = 0.9	-0.004 (-0.09, 0.08), *p* = 0.9	0.01 (-0.07, 0.09), *p* = 0.8	-0.01 (-0.09, 0.06), *p* = 0.7
	Blood group B, 54	0.05 (-0.07, 0.16), *p* = 0.4	0.08 (-0.02, 0.19), *p* = 0.1	0.08 (-0.02, 0.17), *p* = 0.1	0.01 (-0.08, 0.09), *p* = 0.8	-0.01 (-0.09, 0.07), *p* = 0.8
	Blood group AB, 12	0.13 (-0.08, 0.34), *p* = 0.2	0.25 (0.07, 0.44), *p* = 0.008	0.07 (-0.10, 0.24), *p* = 0.4	0.06 (-0.10, 0.21), *p* = 0.5	0.02 (-0.12, 0.17), *p* = 0.8
CD36 (T188G)	Wild type (T/T), 153	0	0	0	0	0
	Heterozygous (T/G), 22	0.08 (-0.06, 0.23), *p* = 0.3	0.03 (-0.10, 0.15), *p* = 0.7	0.03 (-0.08, 0.14), *p* = 0.6	-0.01 (-0.12, 0.11), *p* = 0.9	0.07 (-0.04, 0.18), *p* = 0.2
	Homozygous (G/G), 1	ND	ND	ND	ND	ND
ICAM1 (K29M)	Wild type (K29/K29), 79	0	0	0	0	0
	Heterozygous (K29/M29), 74	-0.03 (-0.13, 0.07), *p* = 0.6	-0.07 (-0.16, 0.02), *p* = 0.1	-0.03 (-0.11, 0.05), *p* = 0.4	0.04 (-0.04, 0.12), *p* = 0.3	-0.06 (-0.14, 0.01), *p* = 0.1
	Homozygous (M29/M29), 23	-0.05 (-0.21, 0.10), *p* = 0.5	-0.01 (-0.15, 0.12), *p* = 0.9	0.06 (-0.06, 0.17), *p* = 0.3	-0.07 (-0.20, 0.05), *p* = 0.2	-0.09 (-0.20, 0.03), *p* = 0.1
HbS	Wild type (HbAA), 198	0	0	0	0	0
	Heterozygous (HbAS), 12	-0.19 (-0.40, 0.01), *p* = 0.07	-0.18 (-0.37, 0.01), *p* = 0.07	-0.08 (-0.25, 0.09), *p* = 0.4	-0.08 (-0.25, 0.08), *p* = 0.3	-0.10 (-0.25, 0.05), *p* = 0.2
	Homozygous (HbSS), 1	ND	ND	ND	ND	ND
Alpha-thalassaemia	Wild type (αα/αα), 77	0	0	0	0	0
	Heterozygous (-α/αα), 106	0.10 (-0.001, 0.20), *p* = 0.05	0.06 (-0.03, 0.16), *p* = 0.2	0.07 (-0.01, 0.15), *p* = 0.08	-0.01 (-0.09, 0.07), *p* = 0.8	0.04 (-0.03, 0.11), *p* = 0.3
	Homozygous (-α/-α), 31	0.03 (-0.11, 0.18), *p* = 0.6	0.02 (-0.11, 0.16), *p* = 0.7	0.03 (-0.09, 0.14), *p* = 0.6	-0.05 (-0.17, 0.06), *p* = 0.3	0.06 (-0.04, 0.17), *p* = 0.2

Results from linear regression models predicting *var* expression levels using the various host polymorphisms are shown. In the case of ABO blood groups, binary variables for blood group A, B and AB were used simultaneously as explanatory variables such that the results are presented relative to blood group O. For the CD36 T188G polymorphism, the ICAM1 K29M polymorphism and alpha-thalassaemia both the heterozygous and homozygous allelic states were used simultaneously as explanatory variables such that the results are relative to the wild type of each host molecule. ND, not done.

## Results and discussion

We used published *var* gene sequence data from 250 isolates obtained from children with clinical malaria (N = 217, of who 111 had severe malaria) or with asymptomatic infection (N = 33) for this analysis ([EMBL accession numbers FN588437–FN592661, HE654181–HE654544]) [7, 14]. The median age of the 217 children with clinical malaria was 3 years (interquartile range (IQR) 1.8 to 4.2) while those with asymptomatic infection had a median age of 5.4 years (IQR 4.1 to 6.7). Neither age nor IE surface antibodies differed between the host polymorphisms considered here (Kruskal-Wallis test *p* > 0.05 for all), with the exception of HbAS, which showed a positive association with IE surface antibodies (Mann–Whitney U test *p* = 0.02) as observed in previous studies [9, 10, 22]. For each host polymorphism Table 1 summarizes the number of children with data available and their distribution. Table 2 provides a comparison of frequencies of each host polymorphism in our dataset with those observed in previous studies in the same geographical setting.

**Table 2:** **Summary of host polymorphism frequencies in the study**

Host polymorphism	Allelic states	Frequency in dataset (%)	Frequency in previous studies (Reference)
ABO blood group	Blood group O	108 (43.2%)	44.7% [23]
	Blood group A	76 (30.4%)	25.5% [23]
	Blood group B	54 (21.6%)	20.2% [23]
	Blood group AB	12 (4.8%)	9.6% [23]
CD36 (T188G)	Wild type (T/T)	153 (86.9%)	83.5% [11]
	Heterozygous (T/G)	22 (12.5%)	15.6% [11]
	Homozygous (G/G)	1 (0.6%)	0.9% [11]
ICAM1 (K29M)	Wild type (K29/K29)	79 (44.9%)	45% [12]
	Heterozygous (K29/M29)	74 (42.0%)	44% [12]
	Homozygous (M29/M29)	23 (13.1%)	11% [12]
HbS	Wild type (HbAA)	198 (93.8%)	91.8% [24]
	Heterozygous (HbAS)	12 (5.7%)	7.7% [24]
	Homozygous (HbSS)	1 (0.5%)	0.5% [24]
Alpha-thalassaemia	Wild type (αα/αα)	77 (36.0%)	35% [25]
	Heterozygous (-α/αα)	106 (49.5%)	48% [25]
	Homozygous (-α/-α)	31 (14.5%)	17% [25]

The proportion of children with each host polymorphism in the present study is compared to proportions observed in previous studies in the same geographical setting and the respective publications indicated.

Whilst the proportion of sampled *var* sequences broadly classified as cys2 showed no association with any host polymorphism, there was evidence for an association between elevated expression of the group A-like cys2 *var* subgroup and blood group AB (Beta = 0.25, 95% CI 0.07, 0.44, *p* = 0.008) but not with blood groups A (Beta = -0.004, 95% CI -0.10, 0.09, *p* = 0.9) or B (Beta = 0.08, 95% CI -0.02, 0.19, *p* = 0.1) (Table 1). However, given the number of comparisons that were performed overall this should be considered preliminary.

**Figure 1:**
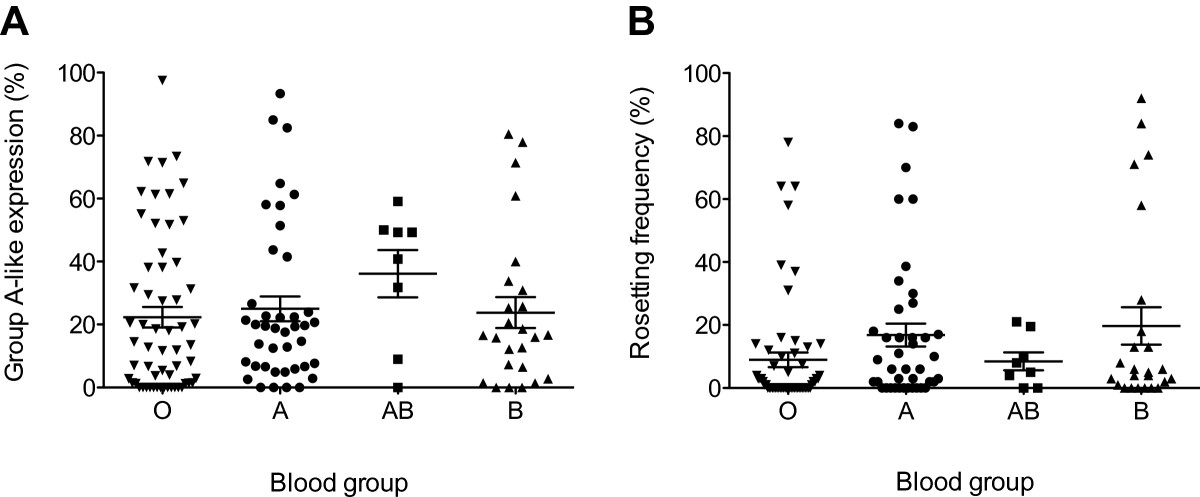
**Parasite group A-like** ***var*** **expression and rosetting in children of different blood groups.** The relationship between parasite group A-like *var* expression and blood group **(A)** and the distribution of parasite rosetting frequencies in isolates from children of different ABO blood groups is shown. The data in both **(A)** and **(B)** represent isolates from each of 133 children whose parasite rosetting data were available, and the mean and standard errors of the mean are shown for each ABO blood group.

Children of blood group A, B or AB tend to be more susceptible to severe malaria than those of blood group O [23, 26]. The association between carriage of the blood group AB and high group A-like *var* gene expression is thus consistent with the fact that parasite isolates from children with severe malaria tend to express higher levels of group A-like *var* genes [7, 27–29]. In our dataset, parasite rosetting, defined as the spontaneous binding of infected to uninfected erythrocytes, was strongly correlated with group A-like *var* expression (N = 133, rho = 0.46, *p* < 0.0001; [14]). However, despite the observed association between blood group AB and high group A-like *var* expression (Table 1), among isolates with rosetting data available (N = 133) parasite rosetting frequency only correlated with blood groups A (Beta = 0.12, 95% CI 0.005, 0.24, *p* = 0.04) and B (Beta = 0.18, 95% CI 0.04, 0.31, *p* = 0.01), but not AB (Beta = 0.04, 95% CI -0.18, 0.25, *p* = 0.7) in a regression analysis adjusted for host age, IE surface antibodies and infection type (also see Figure 1). Though this may seem counterintuitive, the lack of correlation between blood group AB and rosetting is not incompatible with this observation because only a subset of group A *var* genes are known to promote rosetting [30] so it is quite possible that selection for group A PfEMP1 can occur without an increase in the rosetting phenotype. It is therefore possible that, together with age, IE surface antibodies and rosetting, the blood group AB phenotype contributes to the *in vivo* selection pressures that promote group A-like *var* gene expression in the infecting parasite population. To explore this further, we analyzed the relationship again using the same regression analysis framework but instead considering the blood group antigens A and B as individual alleles. In this analysis group A-like *var* gene expression shows evidence of an association with carriage of the blood group B antigen (Beta = 0.12, 95% CI 0.03, 0.21, *p* = 0.008), but the blood group A antigen does not (Beta = 0.03, 95% CI -0.06, 0.11, *p* = 0.5). The sample size was too small to test for an interaction between these two antigens. It is currently unclear what would drive this selection and this observation clearly needs to be verified in further studies.

As shown in Table 1, none of the other host polymorphisms considered here showed an association with detectable alterations in patterns of parasite *var* gene expression, though parasites from HbAS children had somewhat lower expression levels of the group A-like *var* subgroup when compared to those from children with wild type hemoglobin (Table 1). The homozygous states of HbS and the CD36 T188G polymorphism were poorly represented in this dataset (one child each) and hence their effect on parasite *var* gene expression was not formally assessed.

Previous studies have noted the abnormal display of PfEMP1 on the surface of infected erythrocytes from HbAS individuals that correlates with poor endothelial binding properties of such IE, perhaps providing a mechanical basis for the malaria-protective effect of HbAS [31, 32]. In our analysis HbAS children had high IE surface antibody levels (see above) and notably low group A-like *var* expression levels though this latter observation did not reach the conventional level of statistical significance (Beta = -0.18, 95% CI -0.37, 0.01, *p* = 0.07; see Table 1). However, removal of IE surface antibodies as a covariate in the regression modeling strengthened this inverse relationship between HbAS and group A-like *var* expression (Beta = -0.21, 95% CI -0.41, -0.02, *p* = 0.03). The possibility that low group A-like *var* expression levels in HbAS children results from the enhanced IE surface antibody response associated with this host genotype clearly needs further study. Future studies with better host genotype representation also need to consider the poorly understood host-parasite relationship among children with sickle cell disease (HbSS), a major risk factor for death from malaria [24], as well as the known negative epistasis in the malaria-protective effects of HbAS and alpha-thalassaemia [19].

Finally, we predicted an association between parasite *var* gene expression and polymorphisms in ICAM1 and CD36 based on the hypothesis that the reduced *in vivo* concentration of these host ligands resulting from the polymorphisms would give a growth advantage to parasites expression PfEMP1 that can bind to different host ligands. The lack of an association between these polymorphisms and group A-like *var* expression seems inconsistent with previous observations that group A PfEMP1 tend to bind poorly to ICAM1 and CD36 [33, 34]. However our observation is consistent with previous studies in which the cytoadhesion phenotype of parasites sampled from children was compared in individuals with these polymorphisms [35, 36]. Furthermore, a recent study shows that some group A PfEMP1 also bind to ICAM1 making this association less easy to predict [37]. We also note that the proportion of expressed group A-like *var* sequences in the isolate from the only CD36 T188G homozygote in our dataset (12.7%) was below the lower 95% confidence interval of the mean expression levels in isolates from heterozygote (mean = 26.1%, 95% CI 15.4%, 36.9%) and wild type children (mean = 24.1%, 95% CI 20.4%, 27.8%). This may suggest that the effect of this host polymorphism on parasite *var* expression is only evident among homozygotes. Further studies with more T188G CD36 homozygotes are needed to test this.

## Conclusions

In summary, despite sample size limitations for all host factors considered here, our preliminary evidence suggests that host genotype has a potential role in influencing *var* gene expression patterns in the infecting parasite population. More studies with the primary aim of evaluating the effect of host genotype on the whole parasite transcriptome could be a useful approach to validating and understanding mechanisms of host resistance and tolerance to *P. falciparum* infection.

## Authors’ original submitted files for images

Below are the links to the authors’ original submitted files for images. Authors’ original file for figure 1

## Abbreviations

PfEMP1: *Plasmodium falciparum* erythrocyte membrane protein 1
IE: Infected erythrocyte
ICAM1: Intercellular adhesion molecule 1.
