# Supplementary figures and images for: An assessment of the impact of host polymorphisms on Plasmodium falciparum vargene expression patterns among Kenyan children
Source: BMC Infect Dis. 2014 Sep 29;14:524. doi: 10.1186/1471-2334-14-524 (PMC4262213; doi:10.1186/1471-2334-14-524)

**A**

Group A-like expression (%)

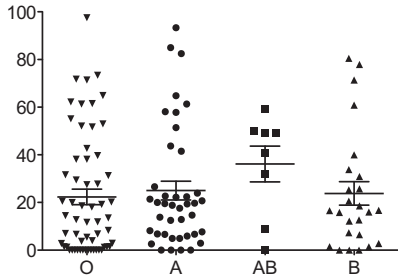

Blood group

**B**

Rosetting frequency (%)

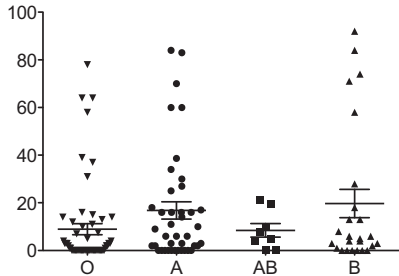

Blood group

Supplement: Supplementary file 1 — Authors’ original file for figure 1 [file 12879_2014_3844_MOESM1_ESM.pdf]
